# The status of CDKN2A alpha (p16^INK4A^) and beta (p14^ARF^) transcripts in thyroid tumour progression

**DOI:** 10.1038/sj.bjc.6603479

**Published:** 2006-11-21

**Authors:** A Ferru, G Fromont, H Gibelin, J Guilhot, F Savagner, J M Tourani, J L Kraimps, C J Larsen, L Karayan-Tapon

**Affiliations:** 1 Laboratoire d'Oncologie Moléculaire EA3805, PBS, Cité Hospitalière de la Milétrie, Avenue du Recteur Pineau 86021, Poitiers, France; 2 Service d'Oncologie Médicale, Poitiers, Cedex, France; 3 Service d'Anatomie Pathologique, Poitiers Cedex, France; 4 Service de Chirurgie Viscérale et Endocrinienne, Poitiers Cedex, France; 5 Centre de Recherche Clinique, CHU-86021 Poitiers Cedex, France; 6 Laboratoire INSERM U694, CHU, 49033 Angers Cedex, France

**Keywords:** P14^ARF^, P16^INK4A^, thyroid, differentiated, carcinomas

## Abstract

CDKN2A locus on chromosome 9p21 encodes two tumour suppressor proteins pl6^INK4A^, which is a regulator of the retinoblastoma (RB) protein, and p14^ARF^, which is involved in the ARF–Mdm2–p53 pathway. The aim of this study was to determine if *CDKN2A* gene products are implicated in differentiated thyroid carcinogenesis and progression. We used real-time quantitative RT–PCR and immunohistochemistry to assess both transcripts and proteins levels in 60 tumours specimens. Overexpression of p14^ARF^ and pl6^INK4A^ was observed in follicular adenomas, follicular carcinomas and papillary carcinomas, while downregulation was found in oncocytic adenomas compared to nontumoral paired thyroid tissues. These deregulations were statistically significant for pl6^INK4a^ (*P*=0.006) in follicular adenomas and close to statistical significance for p14^ARF^ in follicular adenomas (*P*=0.06) and in papillary carcinomas (*P*=0.05). In all histological types, except papillary carcinomas, we observed a statistically significant relationship between p14^ARF^ and E2F1 (*r*=0.64 to 1, *P*<0.05). Our data are consistent with involvement of *CDKN2A* transcript upregulation in thyroid follicular tumorigenesis as an early event. However, these deregulations do not appear to be correlated to the clinical outcome and they could not be used as potential prognostic markers.

Tumours arising from the thyroid follicular epithelium are regarded as a unique model to study human carcinogenesis because, while they all derive from thyrocytes, they have different clinical and histological features (reviewed in Gimm, 2001). Papillary (PTC) and follicular thyroid (FTC) cancers are well-differentiated tumours, which maintain some morphological features of the normal thyroid, and are, most of the time, responsive to radioiodine treatment (Vini and Harmer, 2002). In contrast, anaplastic thyroid cancers are undifferentiated neoplasias with a more aggressive behaviour (Giuffrida and Gharib, 2000). They have lost any significant resemblance with the structural organisation of a normal thyroid follicle, and are unresponsive to both radioactive iodine treatment and chemotherapeutic agents (reviewed in Gimm, 2001). From a molecular standpoint, well-differentiated thyroid cancers are recognised to be initiated by genetic events that involved the improper activation of cellular proto-oncogenes (Pierotti *et al*, 1996). Up to 60% of papillary thyroid carcinomas present either *BRAF* mutations or rearrangements in the *RET* or *NTRK1* tyrosine kinase genes with a plethora of activating genes that are considered to represent the first step in tumour development (Santoro *et al*, 1996; Greco *et al*, 1997, reviewed in Xing, 2005). In 35% of follicular thyroid carcinomas, neoplastic transformation is triggered by activating mutations of the *RAS* gene or by the fusion of the *PAX8* transcription factor with the gene encoding for peroxisome proliferator-activated receptor (Fagin, 2000; Kroll *et al*, 2000). Some of the genetic events responsible for the development of well-differentiated thyroid cancers have been identified; loss of heterozygosity in several chromosomal regions (1p, 3p, 3q, 10q, 11p, 13q and 22q) has been reported in papillary and follicular carcinomas (Ward *et al*, 1998; Kitamura *et al*, 2000a, 2000b). The only established genetic alteration involved in the dedifferentiation process leading to anaplastic thyroid tumour development is the loss of the p53 tumour suppressor gene (Fagin *et al*, 1993).

*CDKN2A* locus on chromosome 9p21 (reviewed in Sherr, 2001) encodes two proteins. INK4A (also referred to as pl6^INK4A^) specially blocks the CDK4 and CDK6 cyclin-dependent kinases that control the phosphorylation status of the retinoblastoma (RB) protein (Serrano *et al*, 1993). ARF (known as p14^ARF^ in man and p19^ARF^ in mouse) has been designed as a positive regulator of p53 levels because, through its complexation with Mdm2 (HDM2 in human), it prevents mdm2-mediated cytoplasmic translocation and degradation of p53 (Kubbutat *et al*, 1997). For this reason, ARF is considered to be an important factor of the so-called ARF–Mdm2–p53 pathway, that is activated by potential oncogenic signals such as oncogenic ras protein, E1A and v-Abl oncoproteins and ectopic expression of both c-myc and E2F1 (Sherr, 1998). Several experimental evidences also unequivocally established that ARF could inhibit cell proliferation independently of p53. For example, introduction of ARF into cells devoid of any functional p53 and Mdm2 proteins results in cell proliferation arrest (Carnero *et al*, 2000; Weber *et al*, 2000). Moreover, p14^ARF^ inhibits the growth of p53 nullizygous human tumours in nude mice and induces the regression of p53−/− established tumours (Eymin *et al*, 2003).

The *ARF* locus is frequently impaired (in conjunction with the INK4A gene) in a variety of human primary tumours, indicating that disruption of this locus is essential for deregulating cell proliferation. However, implication of the p16^INK4A^ and p14^ARF^ has not been clearly defined in thyroid cancer development and progression. The present study was aimed at evaluating possible relationship between the p16^INK4A^ and p14^ARF^ expression on the one hand, and both clinical and pathological features of thyroid carcinomas on the other hand, by using quantitative RT–PCR and immunohistochemistry approaches.

## MATERIALS AND METHODS

### Patients and samples

A total of 102 thyroid tissue samples from 60 patients were processed, including 31 papillary carcinomas, five follicular carcinomas, 10 follicular adenomas, 14 oncocytic adenomas and 42 nontumoral thyroid tissue samples adjacent to carcinomas or adenomas. Tissue samples were obtained from the frozen tissue bank of the pathology department of Poitiers University Hospital. Specimen collection was performed in accordance with the Declaration of Helsinki. Tumour specimens were snap frozen in liquid nitrogen and stored at −80°C until processed for RNA extraction.

All patients were treated at Department of Endocrine Surgery of Poitiers, between 1998 and 2002. They underwent surgical treatment such as unilateral lobectomy, isthmolobectomy or total thyroidectomy according to clinical data. Clinicopathological data of the 60 patients was summarised in Table 1. AGES (age, grade (according to Broder's classification) tumour extent (local invasion and distant metastases) size of the primary tumour) prognostic scores were calculated and patients were included in minimal (score of 0–3.99) or higher risk groups (score of 4 and more) as described by Hay *et al* (1987).

### RNA extraction and cDNA preparation

Total RNAs were extracted from tumour specimens using Qiagen RNeasy® Mini Kit according to the manufacturer's instructions with minor modifications: for exclusion of contaminating genomic DNA, the spin–column membranes were treated with DNase (Qiagen, Courtaboeuf Cedex, France) for 15 min on the bench top before elution. DNase-treated total RNA (3 *μ*g) was transcribed into cDNA using Superscript™ II RnaseH- and random hexamers (Invitrogen).

### Quantitative real-time RT–PCR (QRT–PCR)

We assessed mRNA levels of p14^ARF^, p16^INK4A^, and E2F1 relative to GAPDH in 102 thyroid tissue by using quantitative real time PCR (QRT–PCR) in the ABIPRISM 7000 Sequence Detection System and the human p14^ARF^, p16^INK4a^, E2F1 and GAPDH Taqman Pre-Developed Taqman® Assay Reagents (Applied Biosystems, Foster city, CA, USA). The amplification of p14^ARF^, p16^INK4A^, E2F1 and GAPDH cDNA was carried out in 25 *μ*l reaction volume consisting in 1 × Taqman Universal PCR Master-Mix and 1.25 *μ*l of Pre-Developed Taqman® Assay Reagents (900 nm primers and 200 nM probe) (Applied Biosystems). The reaction was performed as follows: 50°C for 2 min, 95°C for 10 min followed by 40 cycles at 95°C × 15 s, 60°C × 1 min. Each sample was tested in duplicate and a negative control was included in every plate. The computed tomography value was defined as the cycle number (*C*_t_) at which the fluorescence crossed the threshold. The results were expressed in Δ*C*_t_ (*C*_tGAPDH_–*C*_tgene_) or in 2^−Δ*C*_t_^_*_1000 for the amount of the target normalised and relative to the endogenous reference.

### Immunohistochemistry

Immunostaining was performed on 15 papillary carcinomas, five follicular carcinomas, 10 follicular adenomas and nontumoral thyroid tissue samples adjacent to thyroid tumours for each patient. The surgical specimen were fixed in formalin, paraffin embedded and cut in 5 *μ*m tissue sections placed on charged slides. Slides were deparaffinised, rehydrated and microwaved in 10 mM citrate buffer pH 6.0 for antigen retrieval. Immunohistochemistry was performed using mouse monoclonal antibodies directed against p14 ARF (Clone H-132) and p16 INK4A (Clone F12) (Santa Cruz Biotechnology, CA, USA) at 1 : 200 dilution. After blocking for endogenous peroxidase with 1% hydrogene peroxide, the primary antibodies were incubated at 4°C overnight. Immunostaining was performed using the Immunotech automated immunohistochemistry system (Microm Microtech, France). The automated procedure is based on an indirect biotin–avidin system with a universal biotinylated immunoglobulin secondary antibody, diaminobenzidin substrate and haemetoxylin counterstain. Negative controls were obtained by incubation with an immunoglobulin class-matched no immune antibody (mouse monoclonal immunoglobulin, Dakocytomation, Denmark). Positive controls were obtained by immunostaining on reactive lymph nodes, as previously described (Paik *et al*, 2005).

### Scoring of antibody staining

Scoring of antibody staining was performed by pathologist G Fromont who was blind to gene expression status obtained by QRT–PCR. A specimen was considered positive for p14^ARF^ or p16^INK4A^ if there was nuclear staining above any cytoplasmic background. Cytoplasmic staining itself was noted but not included in the scoring (Geradts *et al*, 2001). The immunohistochemical results were evaluated using a semiquantitative analysis: no staining reaction=0, <10% positive-stained cells=+, 10–80% positive cells or focal staining=++, >80% positive cells with diffuse staining=+++.

### Statistical analysis

The data distribution of normalised expression levels of tissue samples is non-Gaussian. So, nonparametric tests were applied for data analysis. The Wilcoxon test was used for comparing tumour tissue to its own nontumoral paired tissue or to mean of the 42 paired tissue expression levels when no paired tissue was available. Upregulation or downregulation was defined by the ratio value between expression level in tumoral tissue and in nontumoral paired tissue (*T*/*S*= 2^−Δ*C*_t_^ tumor/ 2^−Δ*C*_t_^normal tissue). A normal interval was defined for this ratio by the 95% confidence interval of mean for normal tissues and was 0.71–1.32 for p14^ARF^, 0.7–1.40 for p16^INK4A^, 0.85–1.18 for E2F1. For correlations between expression levels and clinicopathological data, the Spearman test was used. *P*-values <0.05 were considered statistically significant.

## RESULTS

### p14^ARF^, p16^INK4A^ expression in thyroid malignancies

**Figure 1:**
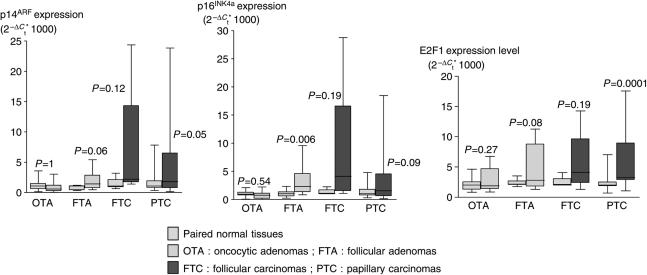
Box plot diagrams showing the expression levels of p14^ARF^, p16^INK4A^ and E2F1. After normalization of sample to their own GAPDH level, data are expressed in 2^−^2^−Δ*C*_t_^_*_1000. OA: oncocytic adenomas; FA: follicular adenomas; FC: follicular carcinomas; PC: papillary carcinomas.

In this work we determined the expression of p14^ARF^ and p16^INK4A^ in 60 thyroid tumours and compared them to their nontumoral paired thyroid tissue control. In all, 18 thyroid tumours without nontumoral paired thyroid tissue control were compared to mean expression of the 42 nontumoral tissues available. We observed an overexpression of p16^INK4A^ and p14^ARF^ transcripts in follicular adenomas, follicular carcinomas and papillary carcinomas and a downregulation in oncocytic adenomas (Figure 1 and Table 2). This overexpression in follicular adenomas was statistically significant for p16^INK4A^ (*P*=0.006) and almost reached statistical significance for p14^ARF^ (*P*=0.06). In follicular carcinomas and oncocytic adenomas, we observed, respectively, an upregulation and a downregulation of p14^ARF^ and p16^INK4A^ expression. Also suggestive, these data were not statistically significant probably because of the too low number of tumour samples analysed (Figure 1 and Table 2). In papillary carcinomas, the expression of p14^ARF^ and p16^INK4A^ was heterogeneous with equal proportions of tumours overexpressing or downexpressing p14^ARF^or p16^INK4A^. The observed deregulation of these two transcripts was close to statistical significance (*P*=0.05 and *P*=0.09) for p14^ARF^ and p16^INK4A^, respectively (Figure 1 and Table 2).

We studied the protein expression level of p14^ARF^ and p16^INK4a^ by immunohistochemistry on papillary carcinomas (*n*=15), follicular adenomas (*n*=10) and follicular carcinomas (*n*=5). Results are summarized in Table 3.

**Figure 2:**
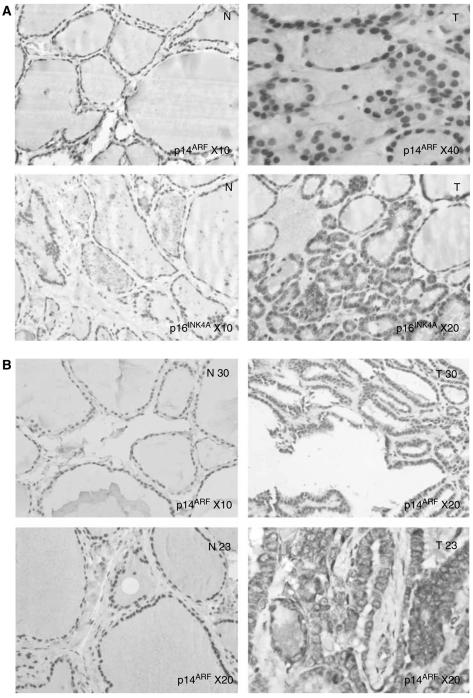
Immunohistochemical detection for p14^ARF^ and p16^INK4A^. (**A**) Follicular adenoma, case 1: Increased p14^ARF^ and p16^INK4A^ expression in tumour (T) when compared to nontumoral tissue (N). (**B**) Papillary carcinoma, case 30: increased nuclear p14^ARF^ expression in tumor (T30) when compared to nontumoral tissue (N30). Case 23: cytoplasmic delocalisation of p14^ARF^ in tumour (T23), when compared to nontumoral (N23). N: nontumoral adjacent tissue, T: Tumour.

In follicular adenomas, p14^ARF^ protein was found to be overexpressed in six cases when compared to nontumoral tissue and p16^INK4A^ in four cases according to QRT–PCR data, (Figure 2A and Table 3). The other cases show identical immunostaining compared to nontumoral tissue. Overexpression of p14^ARF^ and p16^INK4A^ was identified in only one follicular carcinoma, case 13 (Table 3). As in follicular adenomas, the other cases shows identical immunostaining compared to nontumoral tissue. No underexpression of both p14^ARF^ and p16^INK4A^ was observed by immunohistochemistry in follicular tumours.

Among the nine papillary carcinomas that overexpressed p14^ARF^ by QRT–PCR, four showed increased nuclear expression of p14^ARF^ by immunohistochemistry, and interestingly five displayed a cytoplasmic delocalization of the protein (Figure 2B and Table 3). When p16^INK4A^ expression increased by QRT–PCR, immunohistochemical nuclear staining was increased in one case, unchanged in one case, and was localised to the cytoplasm in six cases. Decreased expression of both p14^ARF^ and p16^INK4A^ by QRT–PCR in papillary carcinoma was associated in majority either with decreased or identical immunostaining when compared to normal tissue, or with increased staining in two cases.

In some cases, nodular lymphoid infiltrate was present, mainly in nontumoral tissues, and expressed both p14^ARF^ and p16^INK4A^.

Finally, no significant correlation has been found between p14^ARF^ and p16^INK4A^ mRNA levels and clinicopathological prognostic markers, such as stage, grade, histological subtype, age, node metastasis or AGES score.

### p14^ARF^ regulation by E2F1 is lost in papillary carcinomas

We examined the relationships between mRNA levels p14^ARF^, p16^INK4A^ and E2F1 expression. For this we determined first the mRNA expression levels of E2F1 in the same tumours. Consistent with others studies, E2F1 levels were upregulated in all tumour types (Onda *et al*, 2004). This upregulation was statistically significant in PTC (*P*=0.0001) (Table 2 and Figure 1). A strong correlation occurred between p14^ARF^ and p16^INK4A^ in normal tissue (*r*=0.65, *P*<0.0001) as well as in tumoral tissues (*r*=0.65–0.89 depending on tumour type, *P*<0.05). E2F1 is known to positively regulate p14^ARF^ expression (Bates *et al*, 1998). Indeed, such a relationship was observed between p14^ARF^ and E2F1 expression in normal (*r*=0.41, *P*=0.01) and tumour tissues (*r*=0.64 to 1, *P*<0.05) except for papillary carcinomas (*r*=0.04, *P*=0.86) (Table 4).

## DISCUSSION

The purpose of this study was to analyse p14^ARF^ and p16^INK4A^ mRNA and protein levels in various thyroid tumours including oncocytic adenomas, follicular adenomas, follicular carcinomas and papillary carcinomas. A high prevalence of upregulation for both p14^ARF^ and p16^INK4A^ transcripts was found in follicular adenomas and carcinomas. We observed an heterogenous profiling papillary carcinomas (45% overexpress p14^ARF^ and p16^INK4A^ transcripts and 45% downexpress these transcripts). In the majority of oncocytic adenomas, we observed a downregulation of p14^ARF^ and p16^INK4A^.

We observed a concordance between mRNA and protein levels in most of tumours tested: 80% (24 out of 30) cases for p14^ARF^ and 78.5% (22 out of 28) cases for p16^INK4A^ when cytoplasmic staining observed in papillary carcinomas is taken into account. In 20% of tumors, protein expression did not reflect mRNA status for both p14^ARF^ and p16^INK4A^. This apparent uncoupling have been previously reported on haematopoietic human cell lines (Della Valle *et al*, 1997) and on small-cell lung tumours (Gazzeri *et al*, 1998).

In the majority of the previously documented cases, the discrepancies between mRNA and protein expression corresponds to overexpression of mRNA and normoexpression of the protein. As some patients (particularly cases 4, 12, 15 and 27) showed a high expression of p14^ARF^ and/or p16^INK4A^ in nontumoral tissue, it was difficult to evidence any increase in tumoral tissue. Moreover, the heterogeneity of gene expression within the normal or tumoral tissue must also be taken into account. The presence of a lymphoid infiltrate, which expressed both p14^ARF^ and/or p16^INK4A^, mainly in nontumoral tissue, can also explain some discordant results because positive lymphocytes were not included in IHC scoring. At the end, we should also take into account that accumulation or depletion of transcripts could result from an increased or decreased stability. Regarding this point, impaired mRNA turnover and stability are shown to play a critical role in the activation of specific genes during the cellular response to mitogens, stressful stimuli and differentiation agents (Bashirullah *et al*, 2001; Fan *et al*, 2002). Interestingly, we observed in six cases of papillary carcinoma a cytoplasmic localisation of both p14^ARF^ and p16^INK4A^. This localisation is unusual for these proteins and suggest that in these cases p14^ARF^ and p16^INK4A^ could not be efficients.

Our results are consistent with others studies. Ferenc *et al* (2004) reported such an upregulation by use of a tissue array technology. Intensity of p16^INK4A^ staining tended to increase from follicular adenoma to follicular carcinoma compared to normal tissue. Moreover, others found no mutation and no loss of heterozygosity for the *MTS1* locus (that encodes both p16^INK4A^ and p14^AR^) in differentiated carcinomas (Calabro *et al*, 1996; Schulte *et al*, 1998; Ward *et al*, 1998; Kitamura *et al*, 2000a, 2000b). In contrast, Boltze *et al* (2003) found hypermethylation of *p16*^*INK4a*^ in 33% of follicular adenomas, in 44% of papillary carcinomas, in 50% of follicular carcinomas that correlated with loss of *p16*^*INK4a*^.

Taken together, these data, including our own results, suggest that the inactivation of *p16*^*INK4A*^*/p14*^*ARF*^ locus in these carcinomas is rare and that in contrast, the upregulation of these genes could be associated with thyroid cancer progression.

Both p16^INK4A^ and p14^ARF^ proteins are tumour suppressors and it is their loss not their gain that should contribute to tumorigenesis. However, the increased expression of *CDKN2a* gene products alpha (p16^INK4A^) and beta (p14^ARF^) has been described to be associated with progression and unfavourable prognosis in different tumour types. For instance, the increased expression of p16^INK4A^ has been described to be associated with progression and unfavourable prognosis in ovarian cancer (Dong *et al*, 1997), in high-grade prostatic intraepithelial neoplasia (Henshall *et al*, 2001) and in primary breast cancer (Hui *et al*, 2000). Also, the upregulation of p14^ARF^ is mainly seen in haematological malignancies (Lee *et al*, 2003) and in aggressive B-cell lymphomas, and it predicts a shortened survival time (Sanchez-Aguilera *et al*, 2002).

The overexpression of *p14*^*ARF*^*/p16*^*INK4A*^ tumour suppressor genes could be explained by several known data. Regarding the expression of p16^INK4A^, the most well-defined mechanism of p16^INK4A^ overexpression is the loss of transcriptional repression in the presence of inactivating mutations in the RB gene (Ruas and Peters, 1998). It has been suggested that p16^INK4A^-mediated growth inhibition may occur only when cyclin E/Cdk2 complexes are inactivated concurrently by the cell cycle inhibitor p27^KIP1^(Lukas *et al*, 1997; Jiang *et al*, 1998). In this regard, the MDA-MB-157 breast cancer cell line retains functional pRB but can proliferate in the presence of p16^INK4A^ overexpression (Sweeney *et al*, 1998). Furthermore, others have observed *RAS* and *Rb* mutations in 40–50% of follicular adenomas and carcinomas (Suarez *et al*, 1990; Capella *et al*, 1996; Zou *et al*, 1998) and a downregulation of p27^KIP1^, which was correlated with tumoral progression (Erickson *et al*, 2000; Troncone *et al*, 2000). Although we did not examine *RB* or *Ras* mutations, neither *p27*^*Kip1*^ status in our tumour cohort, these data could explain the p16^INK4A^ upregulation we observed.

Regarding the expression of p14^ARF^, its expression is induced by oncogenes like *myc*, *ras* or viral genes (Palmero *et al*, 1998). E2F1 is a well-known transcriptional regulator of *p14*^*ARF*^ as *p14*^*ARF*^ promoter has an E2F1-binding site (Bates *et al*, 1998). Every cancer-related defect in these pathways should result in p14^ARF^ upregulation. We found an upregulation of E2F1 in all tumour types which is consistent with others studies (Saiz *et al*, 2002; Onda *et al*, 2004), and we observed a link between E2F1 and p14^ARF^ expression in nontumoral thyroid tissue as well as in follicular adenomas and carcinomas. These results suggest the involvement of E2F1 in the p14^ARF^ upregulation in these tumours. The link between E2F1 and p14^ARF^ expression found in nontumoral thyroid tissue was no more present in papillary carcinomas. Strikingly, E2F1 mRNA level was upregulated in all papillary carcinomas, while 45% of them showed a downregulation of p14^ARF^ suggesting that transcriptional regulation of p14^ARF^ in these tumours is independent of E2F1.

Because of their cytoplasmic localisation, these overexpressions of the two proteins are probably nonfunctional. When they are, at first glance, nuclear, their overexpression seems, however, not sufficient to inhibit cell proliferation during the process of thyroid tumorigenesis.

In contrast, in majority of oncocytic adenomas we observed a downregulation of p14^ARF^. As these tumours showed also more frequently decreased expression of E2F1 than other histological types and as a correlation between E2F1 and p14^ARF^ was noted, this suggests that downregulation of p14^ARF^ could be linked to the downregulation of E2F1 in this tumour type.

To conclude, we observed that p14^ARF^ and p16^INK4A^ expression levels are different according to histological type of thyroid tumours. Upregulation seems to be implicated in thyroid follicular carcinogenesis as an early event and could be considered as tumourprogression marker. Papillary carcinomas displayed a more complex pattern with a great heterogeneity for p14^ARF^, p16^INK4A^ expression and an E2F1-independent transcriptional regulation. However, these deregulations are not correlated to the clinical outcome and they could not be used as potential prognostic markers alone.

## Figures and Tables

**Table 1:** Characteristics of patients and tumours

	**All patients**	**Oncocytic adenomas**	**Follicular adenomas**	**Follicular carcinomas**	**Papillary carcinomas**
*n* patients	60	14	10	5	31
Mean age (years)	48.2 (20–92)	48.1 (26–69)	41.6 (20–73)	61.2 (23–92)	48.3 (22–90)
Mean tumour size (mm)	24 (4–100)	30 (12–58)	31 (13–72)	27 (15–40)	19 (4–100)
Multicentricity	17 (23.8%)	8 (57%)	2 (20%)	1 (20%)	6 (19.35%)
Nodal metastasis	—	—	—	1N+ (20%)	3N+ (9.7%)
Juxtathyroid invasion	—	—	—	2 (40%)	6 (19.35%)
Viscéral metastasis	—	—	—	0	0
					
*Tumour grade*					
1	—	—	—	2 (40%)	29 (93.54%)
2	—	—	—	2 (40%)	1 (3.23%)
3	—	—	—	1 (20%)	1 (3.23%)
					
AGES score <4	—	—	—	2 (40%)	26 (83.87%)
AGES score ⩾4	—	—	—	3 (60%)	5 (16.13%)

**Table 2:** Expression levels of p14^ARF^, p16^INK4A^ and E2F1 in nontumoral and in tumoral tissues according to histological type

		**Nontumoral tissue (*n*=42)**	**Oncocytic adenoma (*n*=14)**	**Follicular adenoma (*n*=10)**	**Follicular carcinom (*n*=5)**	**Papillary carcinoma (*n*=31)**
mRNA expression levels	p14^ARF^	1.14 (0.13–9.1)	0.58 (0.025–3.02) (*P*=1)	1.42 (0.45–5.41) (*P*=0.06)	2.20 (1.39–24.35) (*P*=0.12)	1.80 (0.14–23.85) **(*P*=0.05)**
	p16^INK4A^	1.11 (0.05–9.04)	0.73 (0.009–2.21) (*P*=0.54)	2.30 (0.83–9.55) **(*P*=0.006)**	4.10 (1.07–28.75) (*P*=0.19)	1.61 (0.12–52.92) (*P*=0.09)
	E2F1	2.15 (0.68–7.04)	1.89 (0.86–6.75) (*P*=0.27)	2.81 (1.30–11.28) (*P*=0.08)	3.42 (1.03–6.39) (*P*=0.19)	3.24 (1.06–17.58) **(*P*=0.0001)**

The results (medians and minims–maxims) are expressed in 2^−Δ*C*_t_^_*_1000 where Δ*C*_t_=*C*_tGAPDH_–*C*_tgene_. The Wilcoxon test was used to compare the expression levels between nontumoral and tumoral tissues. *P*<0.05 is considered statistically significant. Statistically relevant *P-*values are in bold.

**Table 3:** Immunohistochemical analysis for p14^ARF^ and p16^INK4A^ in normal tissue (N) and thyroid tumours (T) and comparison with their mRNA levels

	**mRNA levels**	**IHC**		**MRNA levels**	**IHC**	
	**p14^ARF^ T/N**	**p14^ARF^ N**	**p14^ARF^ T**	**p16^INK4^ T/N**	**p16^INK4^ N**	**p16^INK4^ T**
*Follicular adenomas*						
Case 1	2.5	+	+++	2.3	++	+++
Case 2	2.5	++	+++	1.4	+++	+++
Case 3	7.5	+	++	9.1	+	++
Case 4	15.5	0	++	11.5	+++	+++
Case 5	0.8	+	+	1.2	+++	+++
Case 6	1.3	++	++	4.2	++	++
Case 7	0.5	++	++	1.3	++	++
Case 8	1.2	++	+++	2.1	++	+++
Case 9	0.7	++	++	0.7	+++	+++
Case 10	3.3	+	++	7.4	+	++
						
*Follicular carcinomas*						
Case 11	1	++	++	0.85	++	++
Case 12	4.2	+++	+++	3.8	+++	+++
Case 13	3.9	++	+++	4	++	+++
Case 14	1.9	+++	+++	1.1	+++	+++
Case 15	22.6	+++	+++	28.3	+++	+++
						
*Papillary carcinomas*						
Case 16	24.3	++	+++	17.2	NA	NA
Case 17	2.6	+	+++	1.2	++	++
Case 18	6.3	+	0 (+C)	2.7	++	0 (+C)
Case 19	3.1	+	+ (+C)	2.1	+	+ (+C)
Case 20	0.5	++	++	0.7	++	++
Case 21	0.1	+++	++	0.1	++	0
Case 22	0.6	++	+	0.2	NA	NA
Case 23	2.2	+++	+ (+C)	1.6	++	+ (+C)
Case 24	0.3	++	+++	0.3	++	+++
Case 25	1.2	++	++	1.7	++	++ (+C)
Case 26	0.3	++	+++	0.5	++	+++
Case 27	9.4	++	+++	25.9	+++	+++
Case 28	5.5	++	++ (+C)	4.8	++	++ (+C)
Case 29	19.3	++	++ (+C)	21.7	++	++ (+C)
Case 30	11.3	0	+++	7.4	++	+++

The immunohistochemical results were evaluated using a semiquantitative analysis: no staining reaction=0, <10% positive-stained cells=+, 10–80% positive cells or focal staining=++, >80% positive cells with diffuse staining=+++. Increased expression appeared in red, decreased expression in green and cytoplasmic localisation in blue. Transcriptional upregulation or downregulation was defined by the ratio value between expression level in tumoral tissue and in normal paired tissue (*T/S*= 2^−Δ*C*_t_^tumour/ 2^−Δ*C*_t_^normal tissue). A normal interval was defined for this ratio by the 95% confidence interval of mean for normal tissues and was 0.71–1.32 for p14^ARF^, 0.7–1.40 for p16^INK4A^ (NA=not analysed).

**Table 4:** mRNA levels correlations between p14^ARF^, p16^INK4A^ and E2F1 genes in nontumoral and in tumour tissues according to histological type

		**Normal tissue**	**Oncocytic adenomas**	**Follicular adenomas**	**Follicular carcinomas**	**Papillary carcinomas**
mRNA level correlations	p14^ARF^ and p16^INK4A^	***r*=0.65**	***r*=0.78**	***r*=0.65**	***r*=0.80**	***r*=0.89**
		***P*<0.0001**	***P*=0.001**	***P*=0.04**	***P*=0.01**	***P*<0.0001**
	p14^ARF^ and E2F1	***r*=0.41**	***r*=0.63**	***r*=0.68**	***R*=1**	*r*=0.04
		***P*=0.01**	***P*=0.015**	***P*=0.029**	***P*<0.0001**	*P*=0.86
	p16^INK4A^ and E2F1	*r*=0.21	***r*=0.7**	*r*=0.48	*r*=0.80	*r*=−0.09
		*P*=0.20	***P*=0.005**	*P*=0.16	*P*=0.1	*P*=0.66

The Spearman rank test was used. The correlation coefficient (*r*) is given with *P*. *P*<0.05 is considered statistically significant. Statistically relevant *P*-values are in bold.
